# Metabotropic Glutamate Receptor 5 Antagonism Reduces Pathology and Differentially Improves Symptoms in Male and Female Heterozygous zQ175 Huntington’s Mice

**DOI:** 10.3389/fnmol.2022.801757

**Published:** 2022-02-02

**Authors:** Si Han Li, Tash-Lynn L. Colson, Khaled S. Abd-Elrahman, Stephen S. G. Ferguson

**Affiliations:** ^1^Brain and Mind Research Institute, University of Ottawa, Ottawa, ON, Canada; ^2^Department of Cellular and Molecular Medicine, Faculty of Medicine, University of Ottawa, Ottawa, ON, Canada; ^3^Department of Pharmacology and Toxicology, Faculty of Pharmacy, Alexandria University, Alexandria, Egypt

**Keywords:** neurodegenerative disease, huntingtin (Htt), G protein-coupled receptor (GPCR), sex differences, striatum, neuroglia, neuronal nuclei (NeuN)

## Abstract

Huntington’s disease (HD) is an inherited autosomal dominant neurodegenerative disorder that leads to progressive motor and cognitive impairment. There are currently no available disease modifying treatments for HD patients. We have previously shown that pharmacological blockade of metabotropic glutamate receptor 5 (mGluR5) signaling rescues motor deficits, improves cognitive impairments and mitigates HD neuropathology in male zQ175 HD mice. Mounting evidence indicates that sex may influence HD progression and we have recently reported a sex-specific pathological mGluR5 signaling in Alzheimer’s disease (AD) mice. Here, we compared the outcomes of treatment with the mGluR5 negative allosteric modulator CTEP (2-chloro-4-[2-[2,5-dimethyl-1-[4-(trifluoromethoxy)phenyl]imidazol-4-yl]ethynyl]pyridine) in both male and female symptomatic zQ175 mice. We found that female zQ175 mice required a longer treatment duration with CTEP than male mice to show improvement in their rotarod performance. Unlike males, chronic CTEP treatment did not improve the grip strength nor reverse the cognitive decline of female zQ175 mice. However, CTEP reduced the number of huntingtin aggregates, improved neuronal survival and decreased microglia activation in the striatum of both male and female zQ175 mice. Together, our results indicate that mGluR5 antagonism can reduce HD neuropathology in both male and female zQ175 HD mice, but sex has a clear impact on the efficacy of the treatment and must be taken into consideration for future HD drug development.

## Introduction

Huntington’s disease (HD) is an inherited autosomal dominant neurodegenerative disease characterized by the early loss of medium spiny neurons in the striatum (Martin and Gusella, 1986). HD symptoms typically manifests between the age of 30–50 and includes choreatic movements, dementia and behavioral difficulties (Roos, 2010). HD is caused by the expansion of a polyglutamine repeat in the N-terminal region of the huntingtin protein (MacDonald et al., 1993). Mutant huntingtin proteins (mHtt) with this expanded polyglutamine repeats have been shown to be targeted for proteolysis and their cleavage at the N-terminus results in the formation of cytoplasmic and intranuclear aggregates that strongly correlate with HD symptoms and severity (DiFiglia et al., 1997). Indeed, longer polyglutamine repeats are associated with earlier disease onset and more severe symptoms (Andrew et al., 1993; Furtado et al., 1996). Despite this well-characterized etiology, disease modifying approaches to treat HD are lacking.

Glutamate is the major mediator of excitatory transmission in the brain and considerable evidence suggests glutamate-induced toxicity and reduction in glutamate uptake contribute to the selective loss of striatal neurons in HD (Hassel et al., 2008; Ribeiro et al., 2011, 2017). Metabotropic glutamate receptor 5 (mGluR5) is a member of the G protein-coupled receptor (GPCR) superfamily that is highly expressed in the striatum and cortex, the two brain regions most affected in HD (Shigemoto et al., 1993; Ribeiro et al., 2017). We have previously reported that mutant but not wildtype huntingtin can disrupt mGluR5 signaling by interacting with it as a part of a protein complex that includes the huntingtin-binding protein optineurin (Anborgh et al., 2005). We have also demonstrated that genetic deletion of mGluR5 in a Q111 mutant huntingtin knock in mouse model reduces mutant huntingtin aggregate size and improves disease pathology (Ribeiro et al., 2014). The prolonged pharmacological blockade of mGluR5 signaling with the negative allosteric modulator CTEP (2-chloro-4-[2-[2,5-dimethyl-1-[4-(trifluoromethoxy)phenyl]imidazol-4-yl]ethynyl]pyridine) also improves HD symptoms and promotes autophagic removal of mutant huntingtin aggregates in the brains of zQ175 HD mouse model (Abd-Elrahman et al., 2017; Abd-Elrahman and Ferguson, 2019). These findings indicate that targeted antagonism of mGluR5 may be effective for the treatment of HD. However, these studies were conducted exclusively in male HD mice and the effects of mGluR5 antagonism on HD pathology in female mice have not yet been investigated.

There is growing evidence that sex may influence HD phenotype and neuropathology in HD rodent models and patients (Dorner et al., 2007; Bode et al., 2008; Zielonka et al., 2013). We recently showed that activation of mGluR2/3 in male and female HD mice led to differential regulation of cell signaling pathways and there are sex-specific differences in cell signaling mechanisms contributing to the pathogenesis of HD (Li et al., 2021). More importantly, we have also reported sex-specific signaling of mGluR5 in AD mice (Abd-Elrahman et al., 2020a; Abd-Elrahman and Ferguson, 2022). Therefore, it is particularly important to study the disease modifying properties of CTEP and assess the contribution of pathological mGluR5 signaling to HD progression in female mice.

Here, we investigated whether targeted antagonism of mGluR5 using CTEP differentially improves HD symptoms and neuropathology in male versus female zQ175 HD mice. We find indeed that chronic treatment with CTEP differentially improves motor and cognitive deficits in male and female zQ175 mice. We also find that CTEP reduces mHtt aggregate pathology, neuronal loss and microgliosis in both male and female zQ175 mice. Our findings point to potential sex-specific differences in the contribution of mGluR5 to HD pathology.

## Materials and Methods

### Reagents

CTEP (1972) was purchased from Axon Medchem (Reston, United States). Rabbit anti-Iba1 (Abcam Cat# ab178847, RRID:AB_2832244) was from Abcam (Cambridge, United States). Mouse anti-NeuN (Millipore Cat# ABN78, RRID:AB_10807945) and anti-Huntingtin clone mEM48 (Millipore Cat# MAB5374, RRID:AB_177645) were from Sigma-Aldrich (St. Louis, MO, United States).

### Animals

All animal experimental protocols were approved by the University of Ottawa Institutional Animal Care Committee and were in accordance with the Canadian Council of Animal Care guidelines. Animals were group caged and housed under a constant 12-h light/dark cycle and food and water were given *ad libitum*. Wildtype and Heterozygous zQ175 HD mice carrying ∼188 CAG repeats were obtained from the Jackson Laboratory and bred to establish littermate-controlled male and female wildtype (Wt) and heterozygous zQ175 (zQ175) mice. Groups of 22 male and female Wt and zQ175 mice were aged to 12 months of age and 11 mice from each group were treated with either DMSO or CTEP (2 mg/kg; dissolved in 10% DMSO and then mixed with chocolate pudding; final DMSO concentration was 0.1%) for 12 weeks. This drug dose was calculated weekly according to weight and was based on our previous studies in male HD mice and AD mice (Hamilton et al., 2016; Abd-Elrahman et al., 2017, 2018, 2020a,2020b; de Souza et al., 2020). All groups were assessed in a battery of behavioral experiments after 4 and 12 weeks of drug treatment. At the end of the 12-week treatment, mice were sacrificed by exsanguination, and the brains were collected and randomized for immunostaining.

### Behavioral Analysis

All animals were habituated in the testing room for a minimum of 30 min before testing. All behavioral tests were performed blindly and during the animal’s dark cycle.

### Forelimb Grip Strength

The grip strength of each mouse was measured using the Chatillon DEF II Grip Strength Meter (Columbus Instruments). Mice were held over the grid of the instrument by their tails and allowed to firmly grip the bar. The mice were then pulled horizontally away from the bar using constant force and at a speed of ∼2.5 cm/s until they released the bar. Each mouse was tested 8 times with a break of 5 s in between each trial and the values of maximal peak force were recorded (Abd-Elrahman et al., 2017).

### Rotarod Test

Mice were introduced to the rotarod apparatus (IITC Life Science, Woodlands Hills, CA, United States) by placing them on the rotarod at rest for 3 min on the first day. Four 5-min-long trails were then performed daily for two consecutive days using an accelerating protocol (from 5 to 45 RPM in 300 s) with 10 min of rest between each trial. Any mice remaining on the rotarod after 300 s were removed and the time scored as 300 s. Average of the latency to fall obtained from the four trials of the second day was used for analysis (Abd-Elrahman et al., 2017).

### Novel Object Recognition

Mice were placed in a square arena measuring 45 cm × 45 cm × 45 cm and tracked using an overhead camera fed to a computer in a separate room. Mice were allowed to explore the empty arena for 5 min, and 5 min later, two identical objects were placed in the arena 5 cm from the edge and 5 cm apart. Mice were returned to the arena for 5 min and allowed to explore. The time spent exploring each object was recorded, and mice were considered exploring the object if their snout was within 1 cm of the object. Twenty-four hours after first exposure, the experiment was repeated with one object replaced with a novel object. The time spent exploring each object was recorded and analyzed using the Noldus EthoVision 10 software. Data were interpreted using the recognition index (time spent exploring the familiar object or the novel object over the total time spent exploring both objects multiplied by 100) and was used to measure the recognition memory [*TA* or *TB*/(*TA* + *TB*)] × 100, where *T* represents the time, *A* represents a familiar object, and *B* represents a novel object (Abd-Elrahman et al., 2017).

### Immunohistochemistry

One hemisphere of each brain sample was fixed in 4%-paraformaldehyde and then transferred to 70% ethanol for storage at 4°C. The samples were embedded in paraffin and then coronally sectioned through the striatum at a thickness of 5 μm. Sections were then incubated with the mouse monoclonal EM48 antibody at 1:100, Neuronal Nuclei (NeuN) antibody at 1:1500, or Iba1 antibody at 1:8000 dilution for 30 min at room temperature and staining was done using Leica Bond III automatic stainer using BOND polymer Refine Detection Kit (Leica Biosystems Cat# DS9800, RRID:AB_2891238) from Leica Biosystems. Slide were scanned using a Leica Aperio Slide scanner at 20× and the number of EM positive aggregates, NeuN or Iba1 positive cells were counted in representative 300 × 300 μm^2^ areas of the striatum. Experimenters were blinded to analysis and six sections per mouse were analyzed and for each section two ROIs in the striatum were quantified using the cell counter tool in ImageJ (Abd-Elrahman et al., 2017, 2020b,2021a; Li et al., 2021).

### Statistical Analysis

Means ± SEM are shown for each independent experiment and *P* < 0.05 was used as the threshold for statistical significance. Statistical significance was assessed using GraphPad Prism 9 software and was determined by Two-way or Three-way Analysis of Variance (ANOVAs) as appropriate, followed by Tukey’s *post hoc* test to determine the source of significant interactions. Statistical details of individual experiments are indicated in figure legends.

## Results

### CTEP Treatment Differentially Rescues Motor Deficits in Male and Female Heterozygous zQ175 Huntington’s Disease Mice

**FIGURE 1:**
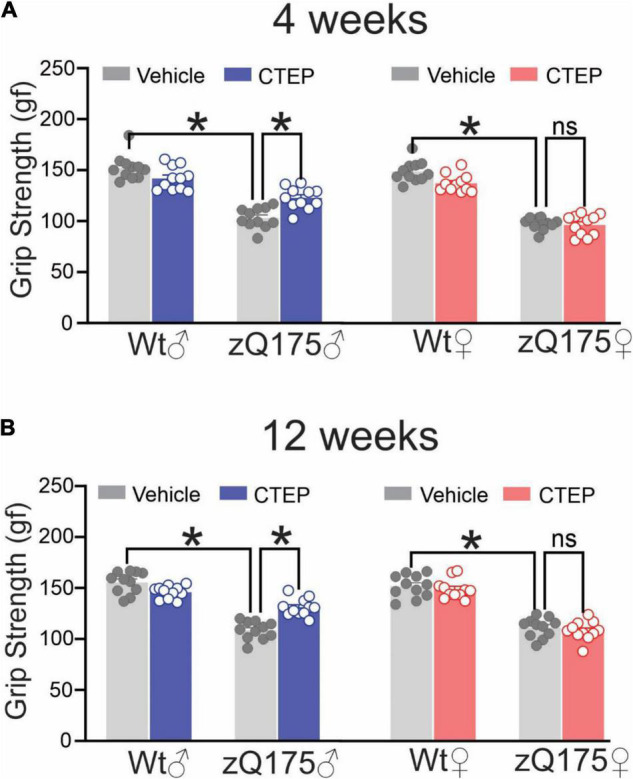
Effect of chronic administration of CTEP on grip strength in male and female zQ175 mice. Mean ± SEM of grip strength [gram-force (gf)] after 4 weeks **(A)** and 12 weeks **(B)** of treatment with either vehicle or CTEP (2 mg/kg/48 h) of 12-month-old heterozygous zQ175 (zQ175) and wildtype (Wt) male and female mice (*n* = 11 for each group). *P < 0.05 by three-way analysis of variance (ANOVA) and Tukey’s multiple comparisons test.

To investigate potential sex-specific differences in the contribution of pathological mGluR5 signaling to HD progression and pathology, we first assessed whether targeted mGluR5 antagonism would improve motor deficits in symptomatic heterozygous male and female zQ175 mice. Twelve-month-old male and female wildtype and heterozygous zQ175 mice were treated with either vehicle or CTEP (2 mg/kg) every 48 h and their motor performance was assessed 4 weeks (13-month-old) and 12 weeks (15-month-old) after the initiation of treatment. The ANOVA indicated that vehicle-treated 13- and 15-month-old male and female heterozygous zQ175 mice showed significant impairment in forelimb grip strength compared to age- and sex-matched, vehicle-treated wildtype mice, *F*’s(1,80) = 343.8 and 323.4; *p* < 0.0001, for 13- and 15-month-old vehicle-treated mice, respectively (Figures 1A,B). After both 4 and 12 weeks of treatment, the forelimb grip strength varied as a function of the Treatment × Genotype × Sex interaction, *F*’s(1,80) = 5.298 and 16.39, *p* = 0.0239 and 0.0001, for 4 and 12 weeks, respectively. The follow-up tests of the simple effects of this interaction confirmed that CTEP treatment for either 4 or 12 weeks led to a statistically significant improvement in grip strength in male but not in female heterozygous zQ175 mice when compared to their sex-matched, vehicle-treated heterozygous zQ175 mice (Figures 1A,B). However, the forelimb grip force of CTEP-treated male heterozygous zQ175 mice remained lower than that of sex- and age-matched, vehicle-treated wildtypes (Figures 1A,B).

**FIGURE 2:**
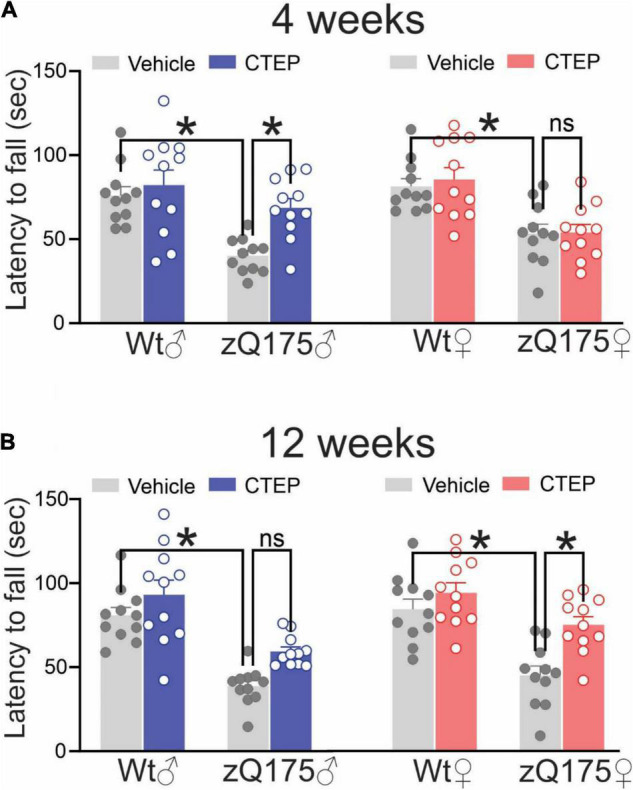
Effect of chronic administration of CTEP on rotarod performance in male and female zQ175. Mean ± SEM of latency to fall (sec) from accelerating rotarod after 4 weeks **(A)** and 12 weeks **(B)** of treatment with either vehicle or CTEP (2 mg/kg/48 h) of 12-month-old heterozygous zQ175 (zQ175) and wild-type (Wt) male and female mice (*n* = 11 for each group). *P < 0.05 by three-way analysis of variance (ANOVA) and Tukey’s multiple comparisons test.

Vehicle-treated (4 and 12 weeks) male and female heterozygous zQ175 mice remained on the rotarod for a shorter time compared to age- and sex-matched, vehicle-treated wildtypes, *F*’s(1,80) = 36.88 and 72.85, *p* < 0.0001, for 13- and 15-month-old vehicle-treated mice, respectively (Figures 2A,B). After 4 weeks of CTEP treatment, the rotarod performance varied as a function of the Treatment × Genotype × Sex interaction, *F*(1,80) = 4.705, *p* = 0.0330. Comparisons of the simple effects of this interaction indicated that 4 weeks of CTEP treatment improved the rotarod performance of male heterozygous zQ175 mice so that their performance was significantly better than that of age- and sex-matched, vehicle-treated zQ175 counterparts (Figure 2A). However, CTEP failed to elicit any significant improvement in female heterozygous zQ175 mice (Figure 2A). A comparable interaction was not apparent after 12 weeks of CTEP treatment, *F*(1,80) = 0.5789, *p* = 0.4490. Specifically, after 12 weeks of CTEP treatment, both male and female heterozygous zQ175 mice showed significantly better rotarod performance relative to vehicle treated heterozygous mice of the same sex, *F*(1,80) = 21.49, *p* < 0.0001 (Figure 2B). In effect, CTEP improved the rotarod performance of both male and female heterozygous zQ175 mice after 12 weeks of treatment, however, the magnitude of this effect was larger in the females than in the males, although the interaction involving this variable was not significant (Figure 2B). Collectively, these data indicated that CTEP treatment differentially rescues motor deficits in female and male heterozygous zQ175 mice and highlight how sex can influence the efficacy of potential drug candidates in reversing specific HD symptoms.

### CTEP Treatment Improves Cognitive Impairment in Male but Not Female Heterozygous zQ175 Huntington’s Disease Mice

**FIGURE 3:**
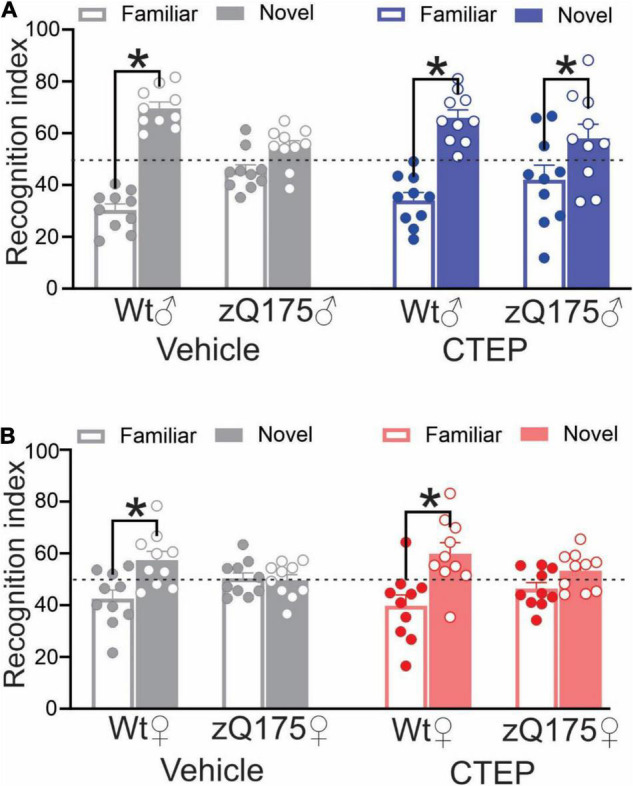
Effect of chronic administration of CTEP on novel object recognition in male and female zQ175 mice. Mean ± SEM of the recognition index, for exploring a novel object versus a familiar object on the second day of novel object recognition test, after 12 weeks of treatment with either vehicle or CTEP (2 mg/kg/48 h) of 12-month-old heterozygous zQ175 (zQ175) and wild-type (Wt) male **(A)** and female **(B)** mice (*n* = 10 for all groups). *P < 0.05 by two-way analysis of variance (ANOVA) and Tukey’s multiple comparisons test.

Huntington’s disease was associated with cognitive impairment in addition to motor deficits (Lemiere et al., 2004). We previously reported that CTEP treatment for 12 weeks improved cognitive impairments in 15-month-old male heterozygous zQ175 mice (Abd-Elrahman et al., 2017). Thus, we assessed whether female heterozygous zQ175 mice exhibited memory impairment in the novel object recognition test at the same age and whether CTEP treatment could alleviate the impairment in female mice. Analysis of the recognition scores revealed a significant interaction between Genotype × Treatment, *F*’s(1,72) = 90.37 and 22.07, *p* < 0.0001 for male and female, respectively. The follow up tests confirmed that unlike wildtypes, where comparable performance was observed irrespective of treatment at 15 months of age, both vehicle-treated male and female heterozygous zQ175 mice failed to distinguish between novel and familiar objects (Figures 3A,B). At the end of the 12 weeks of CTEP treatment, male heterozygous zQ175 mice regained their ability to discriminate between familiar and novel objects but female heterozygous zQ175 mice remained cognitively impaired (Figures 3A,B). Collectively, these data indicated that while both male and female heterozygous zQ175 mice present with cognitive deficits, mGluR5 antagonism does not rescue these deficits in HD mice on a female background.

### CTEP Treatment Reduced Huntingtin Aggregate Number and Neuronal Loss in Both Male and Female Heterozygous zQ175 Huntington’s Disease Mice

**FIGURE 4:**
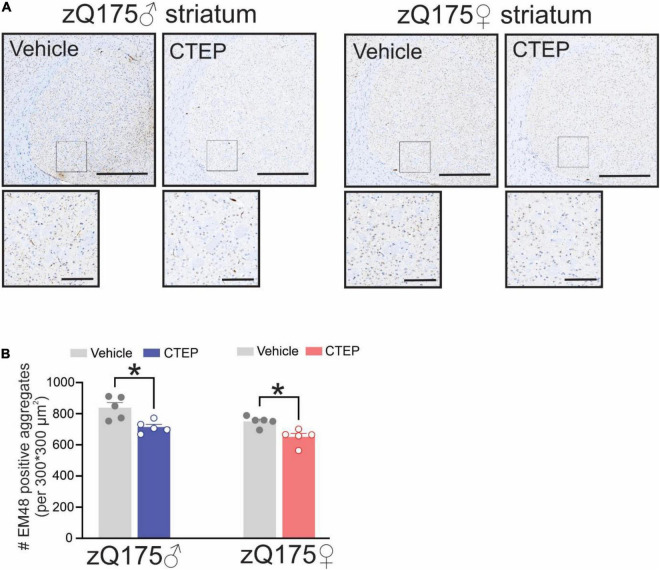
Effect of chronic administration CTEP on mutant huntingtin aggregates in male and female zQ175 mouse striatum. Representative images of staining for mHtt aggregates using the antibody EM48 **(A)** and quantification of the number of huntingtin aggregates **(B)** in striatal brain slices from 15-month-old male and female heterozygous zQ175 mice after 12 weeks of treatment with either vehicle or CTEP (2 mg/kg/48 h). Scale bar = 500 μm for whole striatum and 100 μm for magnified areas. Data are quantified from two different 300 × 300 μm^2^ striatal regions of 6 sections per mouse and five independent mouse brains from each group were used for analysis. Data are mean ± SEM. *P < 0.05 by two-way analysis of variance (ANOVA) and Tukey’s multiple comparisons test.

**FIGURE 5:**
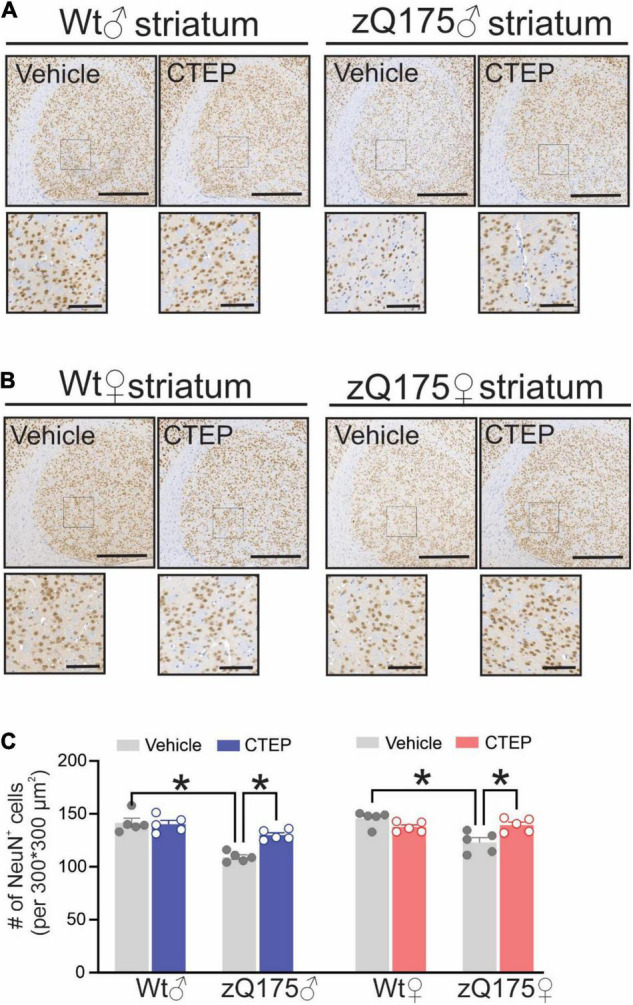
Effect of chronic administration of CTEP on neuronal survival in male and female zQ175 mouse striatum. Representative images of staining for neuronal nuclei (NeuN)-positive cells in striatal brain slices from 15-month-old male **(A)** and female **(B)** heterozygous zQ175 (zQ175) and wild-type (Wt) mice after 12 weeks of treatment with either vehicle or CTEP (2 mg/kg/48 h). Scale bar = 500 μm for whole striatum and 100 μm for magnified areas. **(C)** Quantification of the number of NeuN-positive cells in striatal brain slices from 15-month-old male and female zQ175 and Wt mice after 12 weeks of treatment with either vehicle or CTEP. Data are quantified from two different 300 × 300 μm^2^ striatal regions of 6 sections per mouse and five independent mouse brains from each group were used for analysis. Data are mean ± SEM. *P < 0.05 by three-way analysis of variance (ANOVA) and Tukey’s multiple comparisons test.

The formation of intranuclear and cytoplasmic mHtt aggregates are the pathological hallmark of HD (DiFiglia et al., 1997). We have reported that genetic silencing and pharmacological blockade of mGluR5 reduced the number of mHtt aggregates in male Q111 and zQ175 HD mice, respectively (Ribeiro et al., 2014; Abd-Elrahman et al., 2017). Therefore, we examined whether chronic CTEP treatment can also reduce the number of mHtt aggregates in female zQ175 HD mice. After 12 weeks of CTEP treatment, the number of mHtt aggregates in the striatum of both male and female heterozygous zQ175 mice were significantly reduced compared to age- and sex-matched, vehicle-treated heterozygous zQ175 mice, *F*(1,16) = 23.19, *p* = 0.0002 (Figures 4A,B). Next, we examined whether the improvement in motor function and the decrease in aggregates accumulation were associated with the rescue of neuronal survival. The number of neuronal nuclei (NeuN)-positive cells in the striatum of vehicle-treated 15-month-old male and female heterozygous zQ175 mice was significantly lower than that of age- and sex-matched, vehicle-treated wildtype mice, *F*(1,32) = 48.82, *p* < 0.0001 (Figures 5A–C). Twelve week-treatment with CTEP significantly increased the number of NeuN-positive striatal neurons of both male and female heterozygous zQ175 mice compared to age-and sex-matched, vehicle-treated heterozygous zQ175 mice, *F*(1,32) = 9.361, *p* = 0.0045, and to values that are not different from age- and sex-matched, vehicle-treated wildtype mice (Figures 5A–C). Collectively, these findings indicate that chronic CTEP treatment can reduce HD pathology and rescue neuronal loss in both male and female heterozygous zQ175 mice.

### CTEP Treatment Reduces Microglial Activation in Heterozygous zQ175 Huntington’s Disease Mice

**FIGURE 6:**
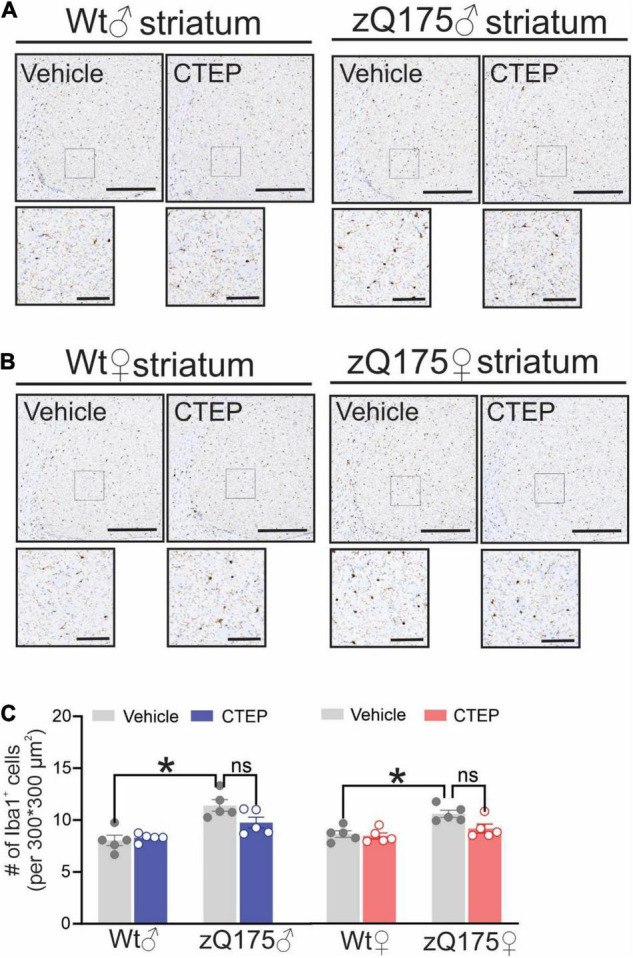
Effect of chronic administration of CTEP on microglia activation in male and female zQ175 mouse striatum. Representative images of staining for ionized calcium binding adaptor molecule 1 (Iba1)-positive cells in striatal brain slices from 15-month-old male **(A)** and female **(B)** heterozygous zQ175 (zQ175) and wild-type (Wt) mice after 12 weeks of treatment with either vehicle or CTEP (2 mg/kg/48 h). Scale bar = 500 μm for whole striatum and 100 μm for magnified areas. **(C)** quantification of the number of Iba1-positive cells in striatal brain slices from 15-month-old male and female zQ175 and Wt mice after 12 weeks of treatment with either vehicle or CTEP. Data are quantified from two different 300 × 300 μm^2^ striatal regions of 6 sections per mouse and five independent mouse brains from each group were used for analysis. Data are mean ± SEM. *P < 0.05 by three-way analysis of variance (ANOVA) and Tukey’s multiple comparisons test.

Microglia activation has been suggested to contribute to the pathogenesis of several neurodegenerative diseases, including AD, Parkinson’s disease, Amyotrophic Lateral Sclerosis and HD (Perry et al., 2010; Abd-Elrahman et al., 2021b). Activation of microglia has been observed in both pre-symptomatic HD gene carriers and symptomatic patients (Pavese et al., 2006; Tai et al., 2007; Björkqvist et al., 2008). Therefore, we assessed the number of activated microglia in the striatum of our mice by staining for ionized calcium-binding adapter molecule 1 (Iba1), a protein that is specifically expressed during microgliosis (Ito et al., 1998). The number of Iba1-positive cells was significantly higher in the striatum of 15-month-old vehicle-treated male and female heterozygous zQ175 mice compared to age- and sex-matched, vehicle-treated wildtypes, *F*(1,32) = 41.48, *p* < 0.0001 (Figures 6A–C). Twelve weeks of CTEP treatment reduced the number of Iba1-positive cells in the striatum of both male and female zQ175 mice, *F*(1,32) = 6.573, *p* = 0.0153, to values that are not significantly different than age- and sex-matched, vehicle-treated wildtypes (Figures 6A–C).

## Discussion

Despite the discovery of its underlying genetic cause decades ago, the exact mechanism(s) underlying HD progression remain poorly understood and treatment options for HD patients are largely symptomatic. Glutamate signaling plays a significant role in the pathophysiology of HD, and the pharmacological blockade of mGluR5 using NAMs delays disease progression in male zQ175 HD mice (Abd-Elrahman et al., 2017). However, several reports have emerged suggesting that sex can influence age of onset and disease progression in HD (Roos et al., 1991; Bode et al., 2008; Zajac et al., 2010; Cao et al., 2018). Furthermore, sex-specific differences in mGluR5 signaling and response to mGluR5 NAMs have been reported in AD mice (Abd-Elrahman et al., 2020a; Abd-Elrahman and Ferguson, 2022). Here, we show that CTEP can indeed improve the performance of male HD mice in all motor and cognitive tasks but fails to elicit similar outcomes in female HD mice. We also demonstrate that CTEP reduces mHtt pathology, microgliosis and neuronal death in both sexes. Our findings point to distinct sex-specific differences in the outcomes of chronic mGluR5 blockade between male and female HD mice that warrants investigating the plausible underlying mechanism(s).

We have previously shown that male heterozygous zQ175 mice at 12 months of age have significant deficits in both motor and cognitive functions that can be reversed by 12-week treatment with CTEP (Abd-Elrahman et al., 2017; Li et al., 2021). Here, we find that both male and female heterozygous zQ175 mice present with significant and comparable impairments in their grip strength and motor coordination that are consistent with previous findings by our group and others using the same mouse model (Menalled et al., 2012; Smith et al., 2014; Abd-Elrahman et al., 2017; Li et al., 2021). We also show that the short (4 weeks) and the long (12 weeks) treatment paradigms are to a great extent equally effective in reversing impairments in grip force and motor coordination of male zQ175 mice during rotarod test. Despite both age-matched male and female HD mice showing deficits in grip strength and motor function, CTEP was not able to improve the performance of female zQ175 mice in most of the motor tasks. Specifically, CTEP did not improve grip strength and only longer treatment with CTEP was able to significantly improve the rotarod performance of female zQ175 mice. Sex-dependent differences in the onset of some motor symptoms were previously reported in another knock-in model of HD, HdhQ350/+ mice (Cao et al., 2018). Moreover, the expression of brain-derived neurotrophic factor (BDNF) was found to be severely affected in female R6/1 mice compared to age-matched males (Zajac et al., 2010). Therefore, it is possible that motor deficits present earlier in female compared to male zQ175 mice and extended treatment is required to reverse these impairments in females. Interestingly, impairment in precision grip control is an early predictor of disease onset and manifest in the pre-HD stage in patients (Rao et al., 2011). Thus, it is also possible that deficits in grip force manifests even earlier than limb coordination and an extended treatment paradigm (beyond 12 weeks) is required to detect a significant improvement in grip strength in female zQ175 mice.

Progressive cognitive decline is another debilitating symptom of HD and MRI study show that HD pathology spreads to the hippocampus, a brain region well-known to be important for learning and memory (Rosas et al., 2003; Lemiere et al., 2004; Bird and Burgess, 2008; van den Bogaard et al., 2011). Additionally, impaired neurogenesis and appearance of mHtt aggregates in the hippocampus have also been reported in animal models of HD (Morton et al., 2000; Simpson et al., 2011; Abd-Elrahman et al., 2017). Here, we show that CTEP reverses cognitive impairment in the novel object recognition test in male but not female zQ175 mice. Interestingly, such an observation is consistent with our most recent work showing that CTEP can reverse deficits in spatial and working memory in male but not female AD mice (Abd-Elrahman et al., 2020a). mGluR5 signaling is differentially regulated between male and female AD mice due to sex-specific differences in the composition of the pathological scaffold formed between amyloid β (Aβ) and mGluR5 (Abd-Elrahman et al., 2020a; Abd-Elrahman and Ferguson, 2022). Thus, it is possible that similar to Aβ, mHtt triggers a sex-specific pathological signaling of mGluR5 that alters the efficacy of mGluR5 NAMs in reversing memory and motor deficits in female HD mice.

Deposition of insoluble mHtt aggregates in the striatum is one of the distinguishing features of HD pathology and mGluR5 is highly expressed in striatum (Shigemoto et al., 1993; DiFiglia et al., 1997). We show that mGluR5 antagonism results in a significant reduction in the number of mHtt aggregates and rescues neuronal loss in the striatum of both male and female zQ175 mice. Since mHtt is known to alter transcriptional regulation and apoptosis (Kim et al., 1999; Rigamonti et al., 2000; Cui et al., 2006), it is likely that the reduction in mHtt following chronic mGluR5 inhibition reduces the loss of striatal neurons and nurture the neurotrophic capacity in HD brains. We have previously reported a similar reduction in apoptotic neuronal loss and mHtt aggregates in male zQ175 mice that was attributed to reactivation of a ZBTB16-dependent autophagy pathway that facilitates the clearance of mHtt aggregates from the striatum (Abd-Elrahman et al., 2017). However, ZBTB16 autophagic pathway is regulated in a sex-specific manner in zQ175 and AD mice and therefore it is likely that the mechanisms underling such reduction in mHtt load and neuronal loss after mGluR5 antagonism are different between both sexes (Abd-Elrahman et al., 2020a; Li et al., 2021). Further investigation in the mechanism(s) underlying such reduction in mHtt pathology in CTEP-treated female zQ175 mice is required in the future.

Activated microglia and elevated levels of pro-inflammatory cytokines have been found in the brains of HD patients and are thought to contribute to HD pathology (Tai et al., 2007; Björkqvist et al., 2008; Silvestroni et al., 2009). mGluR5 is heavily expressed in microglia and the genetic deletion of mGluR5 in BACHD mouse model of HD triggers cortical microgliosis (Biber et al., 1999; Carvalho et al., 2019). We detected microgliosis in the striatum of both male and female zQ175 mice that was abrogated by CTEP, suggesting that mGluR5 antagonism can be effective in reducing neuroinflammation in HD brains of both sexes. It is worth noting that pharmacological silencing of mGluR5 using CTEP in amyotrophic lateral sclerosis (ALS) and AD mice, two neurodegenerative diseases in which glutamate-mediated excitotoxicity plays a crucial role, reduced the number of activated microglia (Abd-Elrahman et al., 2020b; Milanese et al., 2021). Therefore, it is possible that in HD, glutamate excitotoxicity triggers microglial mGluR5 overactivation leading to microgliosis and exacerbation of neuroinflammation that can be abolished by mGluR5 NAMs.

Metabotropic glutamate receptor 5 NAMs remain a promising disease modifying treatment in HD since they are capable of reversing disease pathology in both sexes, but it is possible that extended treatment in females is required to accomplish a significant recovery in motor and cognitive symptoms. The mechanism(s) underlying the sex-specific differences in the efficacy of mGluR5 NAMs in zQ175 HD mice remains unclear. So far, no differences in the subcellular localization, expression, and function of mGluR5 between males and females have been reported in HD. However, mGluR5 can directly interact with mHtt in male Q111 HD mice leading to altered receptor signaling but such interaction has not been investigated in female HD mice (Anborgh et al., 2005; Ribeiro et al., 2010). Furthermore, membrane estrogen receptors are coupled to mGluR5 in female rat striatum and can activate mGluR5 signaling in the presence of estradiol (Grove-Strawser et al., 2010). Therefore, it is possible that mGluR5 interaction with mHtt in HD brain is either intrinsically different between males and females or is influenced by the crosstalk between mGluR5 and sex hormone receptors. Additionally, membrane mGluR5 forms heterodimers and it is possible that the composition of these dimers is regulated in a sex-specific manner leading to differential binding and/or efficacy of mGluR5 allosteric ligands (Prinster et al., 2005; Lee et al., 2020).

## Conclusion

We demonstrate that mGluR5 contributes to HD pathophysiology in male and female zQ175 HD mice and that while mGluR5 NAMs can reverse neuropathology in both sexes, they are less efficacious in reversing symptoms in female compared with male mice. Thus, there are important sex-specific differences in the signaling pathways downstream of mGluR5 that contributes to the pathophysiology zQ175 HD mice that should be investigated in the future. We also emphasize the importance of designing individualized HD treatments that takes both the sex and disease stage of the patient into account.

## Data Availability Statement

The original contributions presented in the study are included in the article/supplementary material, further inquiries can be directed to the corresponding author.

## Ethics Statement

All animal experimental protocols were approved by the University of Ottawa Institutional Animal Care Committee and were in accordance with the Canadian Council of Animal Care guidelines.

## Author Contributions

SL, KA-E, and SF were responsible for the conception and design of all experiments. SL, KA-E, and T-LC performed the experiments and data analysis. SL and KA-E wrote the manuscript. SF edited the manuscript and supervised the study. All authors contributed to the article and approved the submitted version.

## Conflict of Interest

SF holds a Tier I Canada Research Chair in Brain and Mind. KA-E is a Lecturer at the Department of Pharmacology and Toxicology, Faculty of Pharmacy, University of Alexandria, Egypt. The remaining authors declare that the research was conducted in the absence of any commercial or financial relationships that could be construed as a potential conflict of interest.

## Publisher’s Note

All claims expressed in this article are solely those of the authors and do not necessarily represent those of their affiliated organizations, or those of the publisher, the editors and the reviewers. Any product that may be evaluated in this article, or claim that may be made by its manufacturer, is not guaranteed or endorsed by the publisher.

## Abbreviations

AD: Alzheimer’s disease
HD: Huntington’s disease
Iba1: ionized calcium binding adapter molecule 1
mGluR: metabotropic glutamate receptor
mHtt: mutant huntingtin
NeuN: neuronal nuclei
NMDAR: N-methyl -D-aspartate receptor
PKC: protein kinase C
PLC: phospholipase C.
